# Editorial: highlights in physical activity in the prevention and management of disease 2021/22

**DOI:** 10.3389/fspor.2023.1207393

**Published:** 2023-05-02

**Authors:** John G. Morris, Carlos A. Celis-Morales

**Affiliations:** ^1^ Department of Sport Science, Nottingham Trent University, Nottingham, United Kingdom; ^2^ School of Cardiovascular and Metabolic Health, University of Glasgow, Glasgow, United Kingdom

**Keywords:** COVID-19, health, performance, exercise, workers, lower back pain

**Editorial on the Research Topic** Highlights in physical activity in the prevention and management of disease 2021/22

Humans are meant to be active. Or, at the very least, during their evolution they were not able to be inactive and hence many of our underlying adaptations to inactivity can result in metabolic and mental dysfunction. The selection of papers in this special issue (described in brief below) are drawn from a wide variety of sources and seek to understand how physical activity can be used to prevent and manage disease across the lifespan.

A number of the papers in this special issue examined the impact of the COVID-19 pandemic on physical activity and sedentary behaviour. Schmidt and Pawlowski examined the effect of the COVID-19 shutdown on the amount of leisure-time physical activity undertaken by 1,800 Danish citizens aged 15–18, 19–29, 30–59 and 60+ years old, using a cross-sectional survey methodology. They found that, compared to before the shutdown, the proportionate decrease in mean minutes of leisure-time physical activity per week was greatest in the teenage group (−36.6%), followed by the oldest and young adult groups (by −24.9% and −21.3% respectively). The authors noted that although undertaken to prevent disease spread, the shutdown reduced leisure-time physical activity substantially, and ultimately this could have serious public health consequences. In their study, Sugano et al. conducted an internet-based survey of 27,000 Japanese workers during the COVID-19 pandemic, which examined the relationship between their exercise and physical activity habits and their self-assessed, health-related quality of life. Compared to workers who “almost never” undertook any exercise or physical activity, those who took at least some had better self-assessed health. Interestingly, the beneficial effects of exercise were still evident when the outcome was the number of “physically or mentally unhealthy days” in the last 30, but no beneficial effect was evident for physical activity. The authors concluded that even short bouts of daily exercise could have beneficial effects on workers health and work performance, particularly in workers who almost never undertook exercise. Morton et al., using a review of 22 studies based on a systematic search, sought to identify ways in which sedentary behaviour could be reduced in an office environment. Also, given the post COVID-19 culture of increased home working, they sought to examine which of the identified approaches might have utility in the home working environment. In the office, interventions based on environmental restructuring, training, enablement and education, often used in combination, were the most common approaches taken to reduce sedentary behaviours, but not all would be easily transferable to home working situations.

Being sedentary often involves long periods of sitting. The study by Kett et al. showed that neuromuscular stimulation of the lumbar spine during 4.5 h of sitting significantly reduced back muscle stiffness. The authors noted that their research helps explains why long periods of chair sitting without muscular activity may increase the likelihood of lower back pain. Their study also emphasises why consistent muscle activity throughout the day is really important if common but debilitating conditions such as lower back pain are to be attenuated, and perhaps prevented.

The ability to measure and monitor characteristics such as maximal oxygen uptake (cardiorespiratory fitness) and motor competence are really important. “Cardiorespiratory fitness” is associated with life expectancy, mortality and performance, and so it is an important metric in a wide variety of situations and groups. Typically, it is determined using directly measured oxygen uptake during a maximal test. However, in those with clinical dysfunction maximal efforts may not be possible and, even if they are, may be associated with unacceptable risks. Eike et al. evaluated a modified Blake protocol for assessing maximal oxygen consumption and found that the modified protocol was valid compared to directly determined values (*R*^2 ^= 0.78; SEE = 3.1 ml·kg^−1^·min^−1^; *p* < 0.001), and that the modified protocol was feasible for use in individuals at high risk or with chronic health conditions. Drenowatz et al. argue there is linkage between motor competence and physical fitness, a physically active lifestyle and ultimately future public health. In their study they report the preliminary findings from a state wide fitness testing program that examined cardiorespiratory fitness, muscular power, speed, agility, flexibility, object control skills, stature and body mass, and involved 18,000 six- to eleven-year-old Austrian school children. They noted that, while there were expected improvements in fitness and motor competencies as age increased, in those participants designated overweight or obese, physical fitness (except for upper body strength) and motor competence were lower than in normal weight groups. A key issue seemed to be the ability of individuals who were not normal weight to move their own body mass, and while the normal weight group showed improvement in their 6 min run time as they aged, there was no evidence of such improvement in the overweight/obese group.

The importance of involvement in organised sports in childhood and adolescence on future quality of life is also an interesting question. Appelqvist-Schmidlechner et al. examined the association between sports participation and mental and physical components of health-related quality of life using a cross-sectional design, a survey methodology and 777 young adult Finnish males. The study noted that participation in organised sports at age 12 was mainly positively associated with the mental component of their health-related quality of life outcome, rather than the physical component. They also found that a greater involvement in organized sports in childhood increased the likelihood of a higher health related quality of life in young adulthood. The authors noted that their study emphasised the importance of encouraging and facilitating opportunities for organised sports participation in all children and young people.

Other studies in this special issue performed interventions to examine how exercise alone or exercise in combination with nutritional and psychological guidance in middle-aged or adolescent populations influenced markers of cardio-respiratory fitness and health. De Borba Schneiders et al. performed a quasi-experimental study in which seventeen participants (aged 10–17 years-old) were allocated to an intervention group who undertook a 6-month multi-component program comprised of a variety of types of exercise, and nutritional and psychological guidance. Twenty individuals with similar characteristics were allocated to a control group. The study aimed to verify that such a program could positively impact physical fitness, body composition and markers of insulin secretion and resistance in overweight and obese adolescents. While the program improved some body composition outcomes (e.g., body fat percentage) and cardiorespiratory fitness (6 min walking/run test), it had no measurable effect on insulin or HOMA-IR. Collins et al. examined how three different 8-month exercise interventions (aerobic training, resistance training, or aerobic and resistance combined) influenced health-related quality of life (determined by survey responses) in 137 middle-aged women and men (49.0 ± 10.6 years). They found that all of the exercise programmes had some impacts on self-reported health-related quality of life components, but all groups showed significant improvements in peak oxygen uptake, and satisfaction with physical function and appearance scores.

All the studies in this special issue demonstrate the wide variety of important research that is being conducted to understand how physical activity can be used to prevent and manage disease across the lifespan.

